# Supplementary material for: PglZ from Type I BREX phage defence systems is a metal-dependent nuclease that forms a sub-complex with BrxB
Source: Nucleic Acids Res. 2025 Jun 23;53(12):gkaf540. doi: 10.1093/nar/gkaf540 (PMC12205998; doi:10.1093/nar/gkaf540)
Supplement: gkaf540_Supplemental_File [file gkaf540_supplemental_file.pdf]

Supplementary Material

PglZ from Type I BREX phage defence systems is a metal-dependent nuclease that forms a sub-complex with BrxB

Jennifer J. Readshaw[a,*], Lindsey A. Doyle[b,*], Maria Puiu[a], Abigail Kelly[a], Andrew Nelson[c], Alex J. Kaiser[b], Sydney F. McGuire[d], Julieta Peralta Acosta[d], Darren L. Smith[c], Barry L. Stoddard[b], Brett K. Kaiser[d,†], Tim R. Blower[a,e,†]

[a]Department of Biosciences, Durham University, Stockton Road, Durham, DH1 3LE, UK.

[b]Division of Basic Sciences, Fred Hutchinson Cancer Center, 1100 Fairview Ave. N. Seattle WA 98019, USA.

[c]Department of Applied Sciences, University of Northumbria, Newcastle Upon Tyne NE1 8ST, UK.

[d]Department of Biology, Seattle University, 901 12th Ave. Seattle WA 98122, USA.

[e]New England Biolabs, 240 County Road, Ipswich, MA 01938, USA.

[*]These authors contributed equally.

[†]To whom correspondence may be addressed. Email: kaiserb@seattleu.edu, timothy.blower@durham.ac.uk, tblower@neb.com.

Keywords: Phage defence, Bacteriophage exclusion, nuclease, metal-dependent, BREX

Supplementary Figure S1

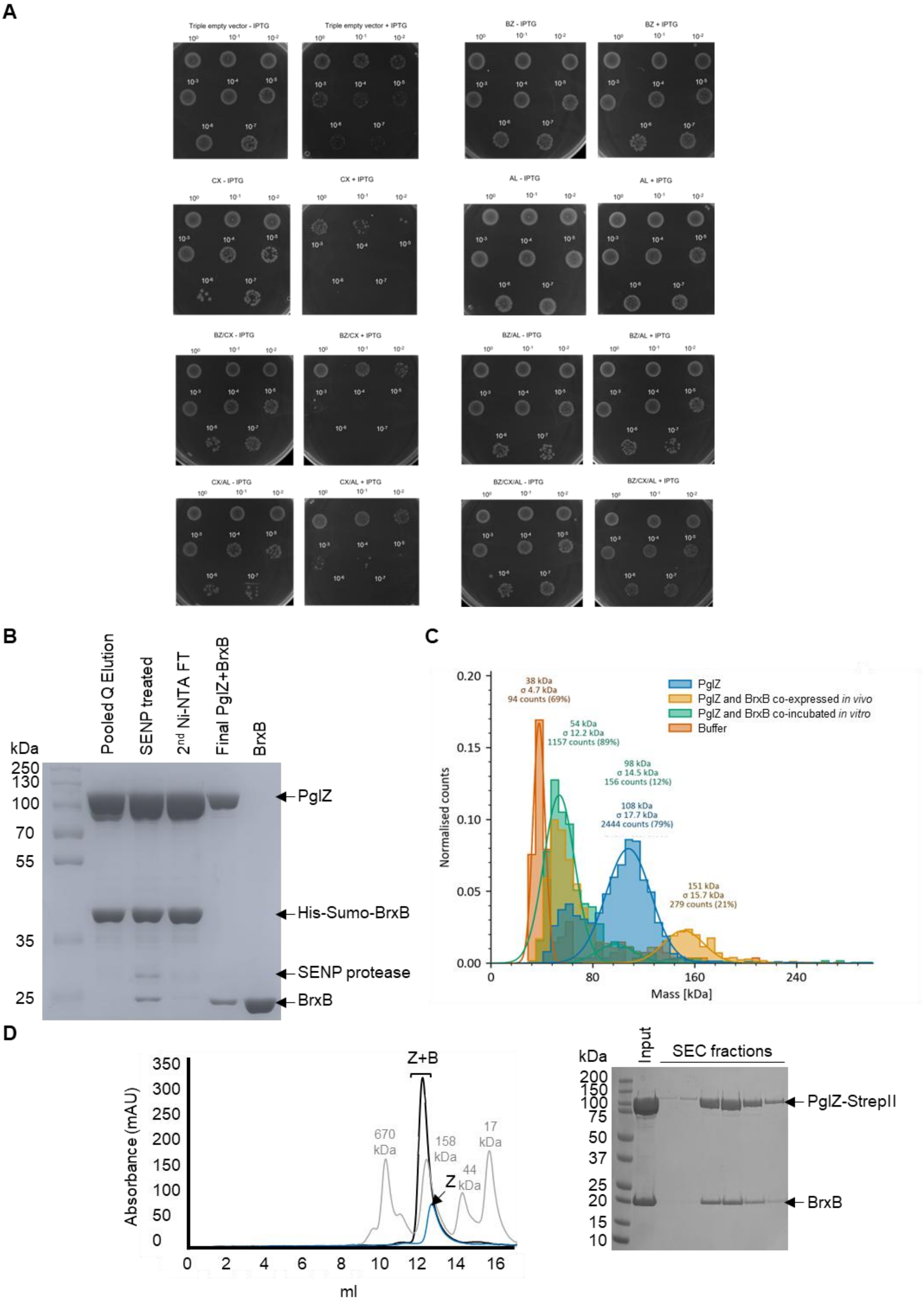

Supplementary Figure S1. BREX protein toxicity tests and purification of PglZ:BrxB complexes. (**A**) Spot tests for toxicity during expression of *Salmonella* BREX proteins. Plates are representative of triplicate data. (**B**) Selected samples during production of *Salmonella* PglZ:BrxB complexes for structural study. (**C**) Mass photometry analysis of *Salmonella* PglZ:BrxB samples. (**D**) Size exclusion chromatography traces for *Acinetobacter* PglZ (blue) and PglZ:BrxB (black), with molecular weight standards shown in grey. Fractions for the PglZ:BrxB purification are shown separated by SDS-PAGE.

Supplementary Figure S2

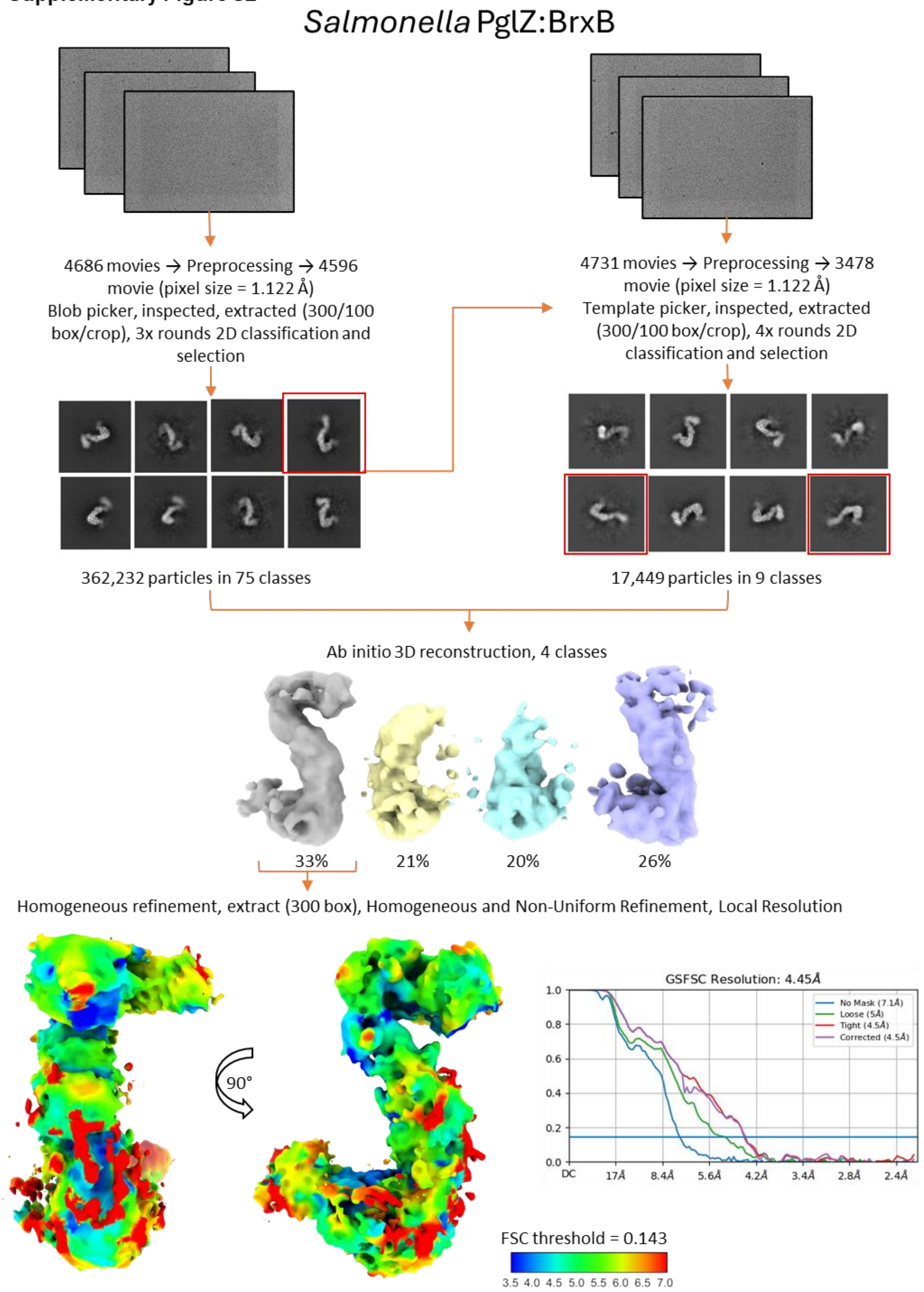

Supplementary Figure S2. Data processing summary of the *Salmonella* PglZ:BrxB complex. A small subset of the total 2D classes is shown, with deviation from the "S" shape highlighted with red boxes.

Supplementary Figure S3

Orientation Diagnostics of *Salmonella* PglZ:BrxB

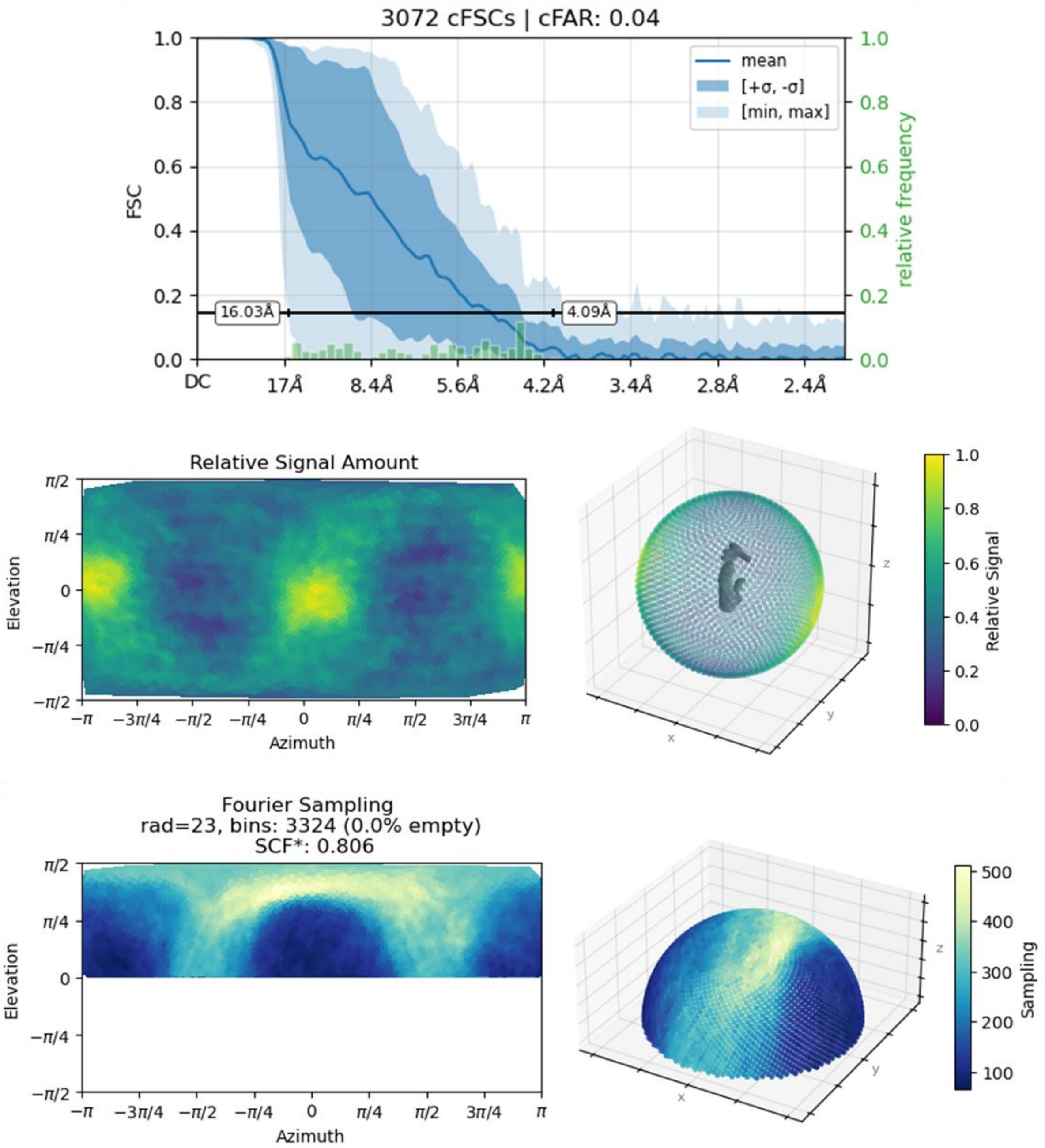

Supplementary Figure S3. CryoSparc Orientation Diagnostics output. The data gives a conical FSC area ratio (cFAR) of 0.04, indicating intense preferred orientation.

Supplementary Figure S4

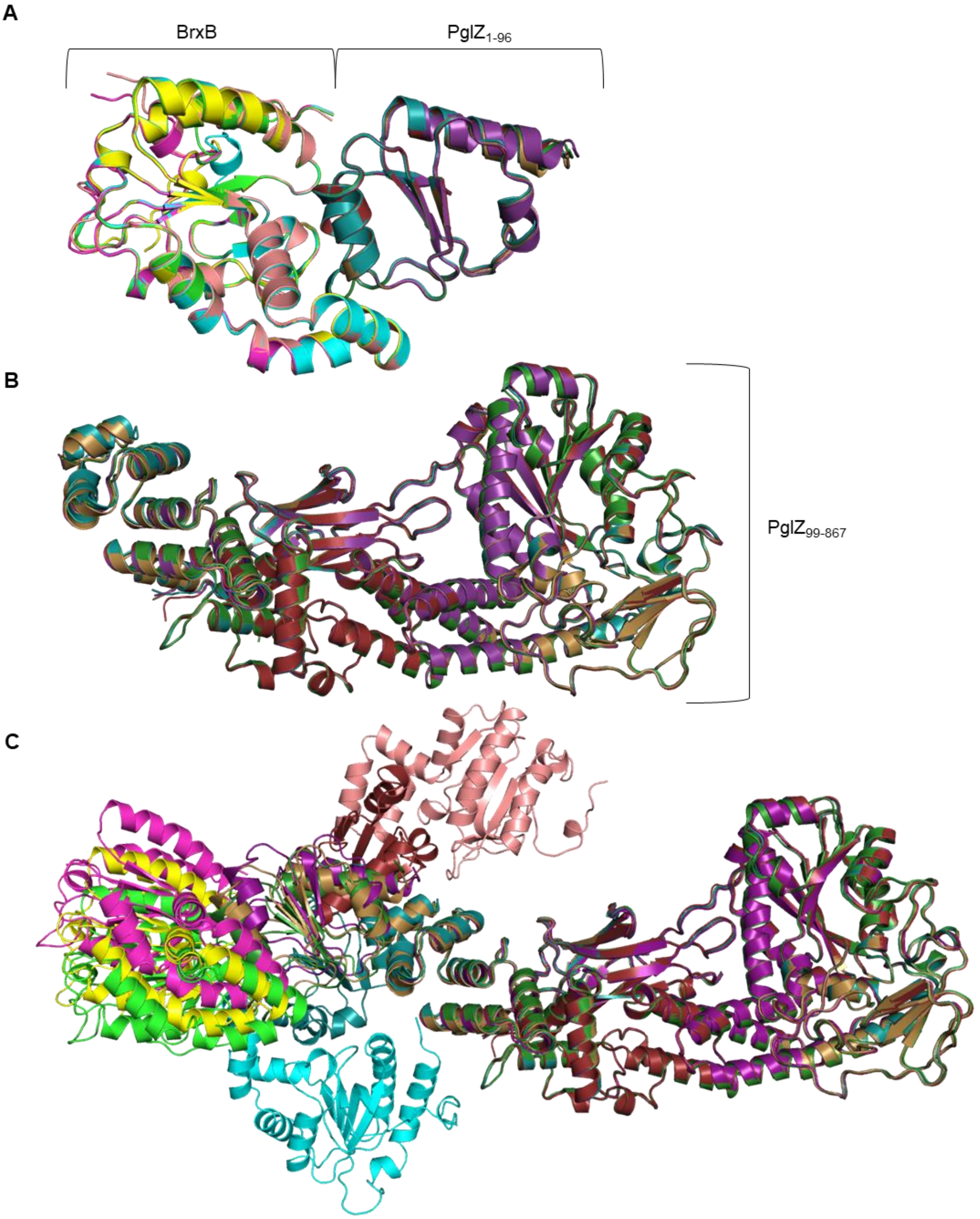

Supplementary Figure S4. Alignment of five AlphaFold3 models of *Salmonella* PglZ shows a flexible N-terminal domain that allows movement of BrxB. (**A**) BrxB:$PglZ_{1\text{-}96}$ gives an average C-alpha RMDS = 0.16 Å. The variation is largely concentrated in the ten C-terminal residues of BrxB. (**B**) $PglZ_{99\text{-}867}$ gives an average C-alpha RMSD = 0.48Å. (**C**) Full BrxB:PglZ complex aligned against $PglZ_{99\text{-}867}$ shows variable locations of BrxB:$PglZ_{1\text{-}96}$ indicating a hinge point between residues 96-99 in PglZ.

Supplementary Figure S5

A

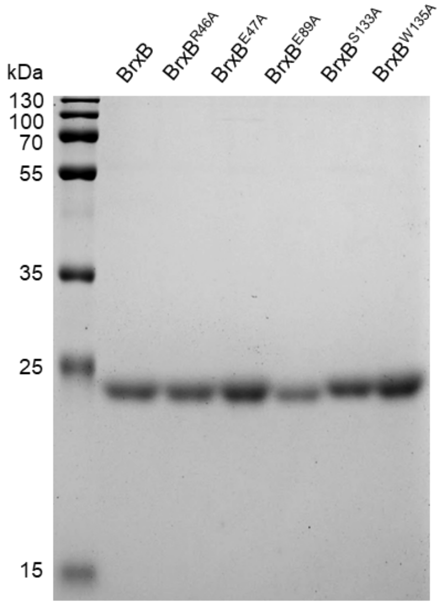

B

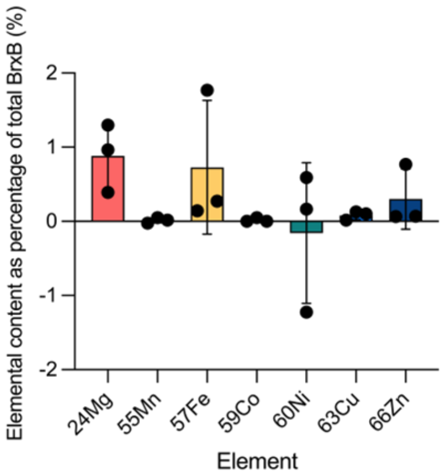

C

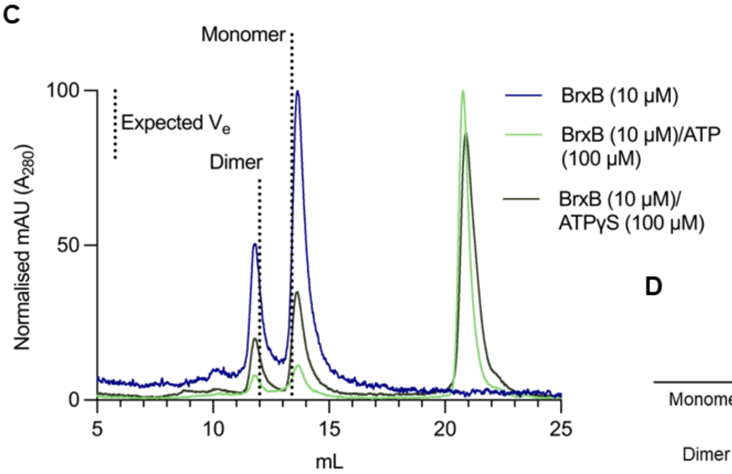

D

	Calculated Rst (Å)	Observed Rst (Å)	Observed Rst (Å)/ Calculated Rst (Å)
Monomer	23.7	23.3	0.98
Dimer	29.1	30.2	1.04

E

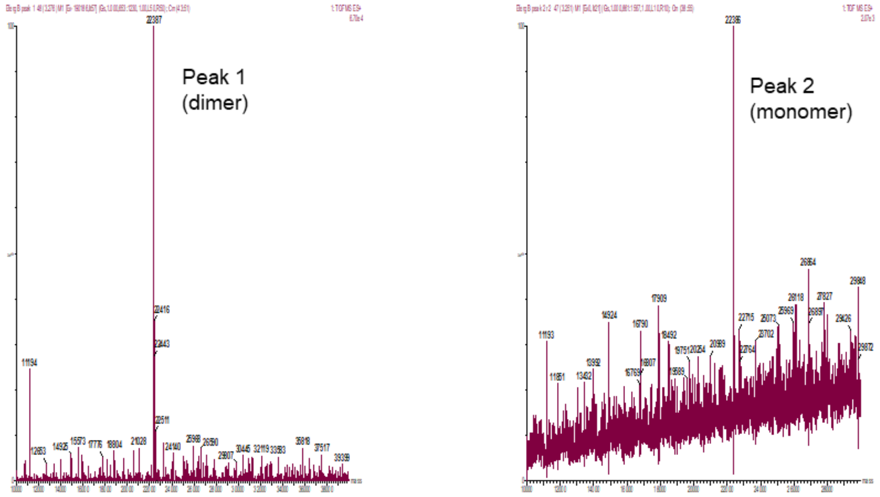

Supplementary Figure S5. *E. fergusonii* BrxB does not bind metals and exists as both dimers and monomers. (**A**) SDS-PAGE of purified *E. fergusonii* BrxB WT and mutants loaded at 4 µg on a 12% (v/v) polyacrylamide gel. Samples were resolved for 1 h 15 min in tris-glycine running buffer and stained with Quick Coomassie. (**B**) ICP-MS of BrxB WT and mutants showing divalent cations bound following purification. Plotted data represent mean values ± SD. Metal content is plotted as a percentage of the total protein in the sample. (**C**) Analytical SEC (S75i) of BrxB WT (10 µM) with and without nucleotides and comparison against expected elution (V_e) using Stokes radii. (**D**). The expected elution volumes (V_e) of monomeric and dimeric BrxB are represented by black dotted lines. (**E**) Electrospray ionisation mass spectrometry of 'monomeric' and 'dimeric' SEC peaks shows BrxB is present in both peaks.

Supplementary Figure S6

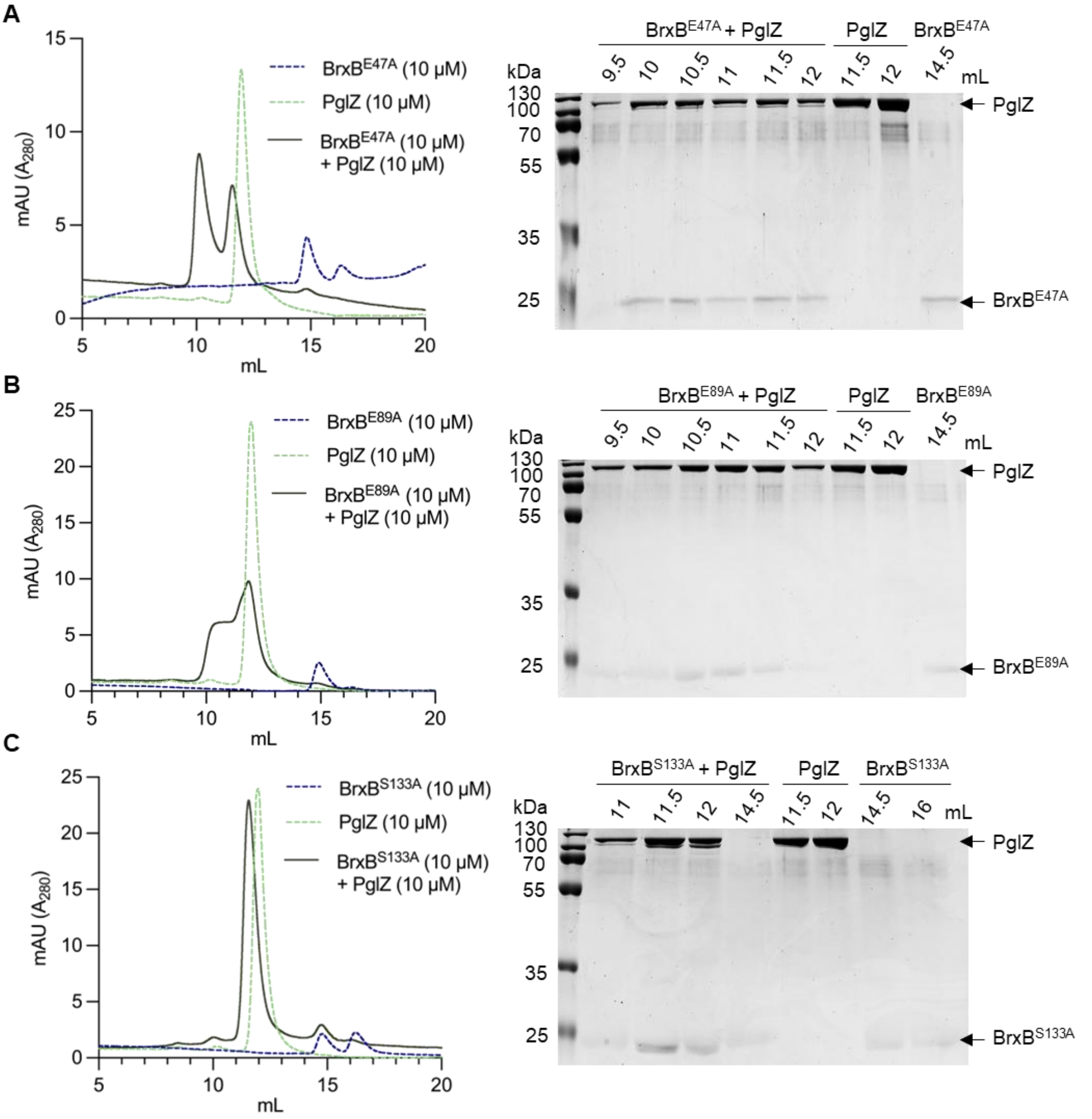

Supplementary Figure S6. Analytical SEC (S200i) and SDS-PAGE analysis of *E. fergusonii* PglZ and BrxB mutants. PglZ forms complexes with BrxB mutants E47A (**A**), E89A (**B**), and S133A (**C**). Samples of PglZ (10 µM) with equimolar BrxB WT and mutants were made up to 100 µl and pre-incubated for 15 min prior to loading on the S200i. The elution profile of PglZ incubated with BrxB is shown as a dark green solid line. Control elution profiles of PglZ alone (light green dashed line) and BrxB alone (dark blue dashed line) are also shown. Fractionated peak samples were resolved on 15% (v/v) polyacrylamide gels for 1 h 15 min in tris-glycine running buffer and stained with Quick Coomassie. Protein identities are highlighted with black arrows.

Supplementary Figure S7

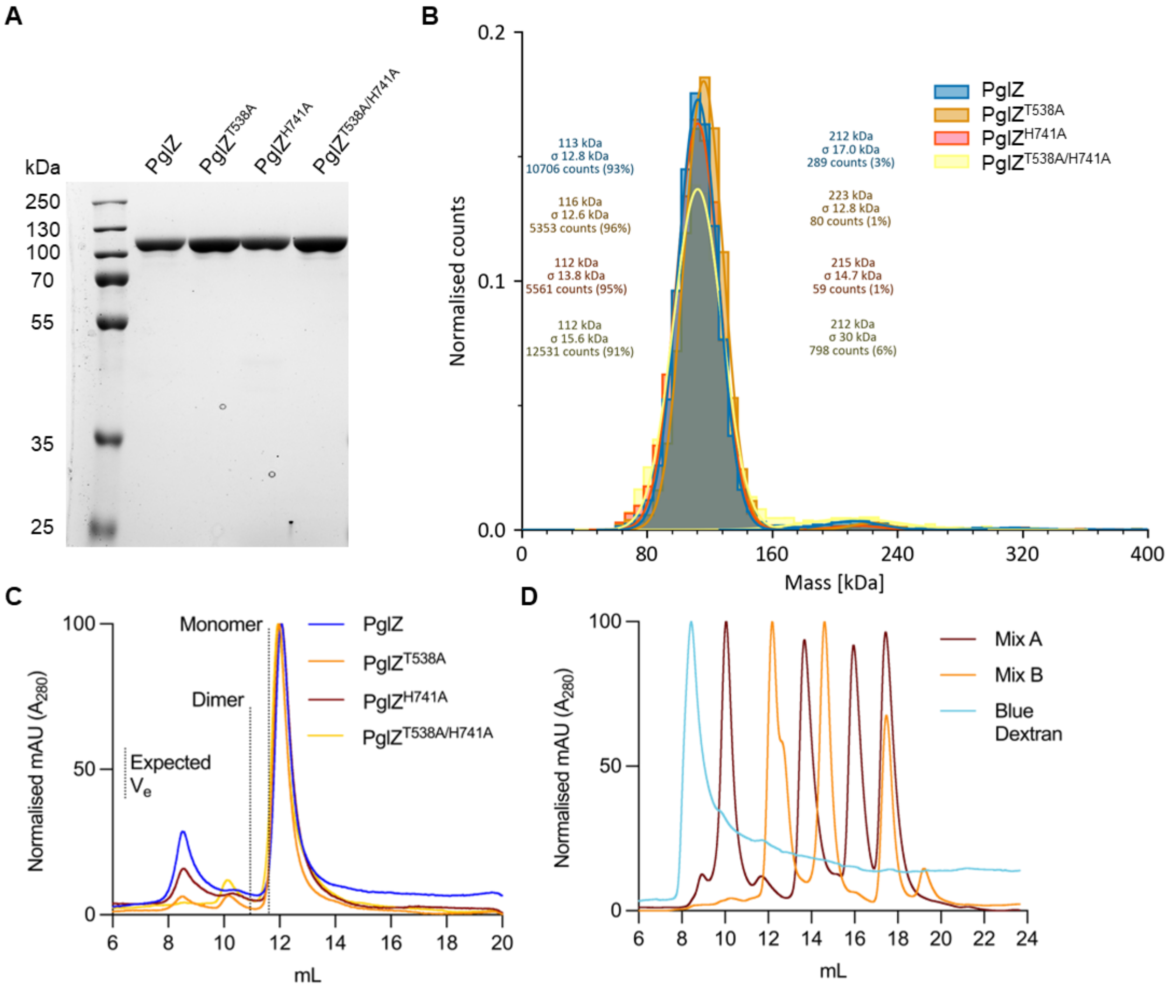

E

	Calculated Rst (Å)	Observed Rst (Å)	Observed Rst (Å)/ Calculated Rst (Å)
Monomer	50.11	46.70	0.93
Dimer	56.00	62.46	1.12

Supplementary Figure S7. *E. fergusonii* PglZ WT and mutant production. (**A**) SDS-PAGE of PglZ WT and mutants loaded at 4 µg on 15% (v/v) polyacrylamide gel. Samples were resolved for 1 h 15 min in tris-glycine running buffer and stained with Quick Coomassie. (**B**) Mass photometry of PglZ WT and mutants. Counts were acquired for 60 s with 5 nM protein samples in PBS. (**C**) Analytical SEC (S200i) of PglZ WT and mutants. Protein samples were loaded at 10 µM in a 100 µl final volume. The expected elution volumes (V_e) of monomeric and dimeric PglZ are represented by black dotted lines. (**D**) Calibration of analytical SEC S200i column using protein standards. Mix A: RNase A, Ferritin, Conalbumin, and Carbonic anhydrase. Mix B: RNase A, Aldolase, Ovalbumin, and Aprotinin. (**E**) Comparison of expected elution points based on Stokes radii. Presented data are representative of triplicate data.

Supplementary Figure S8

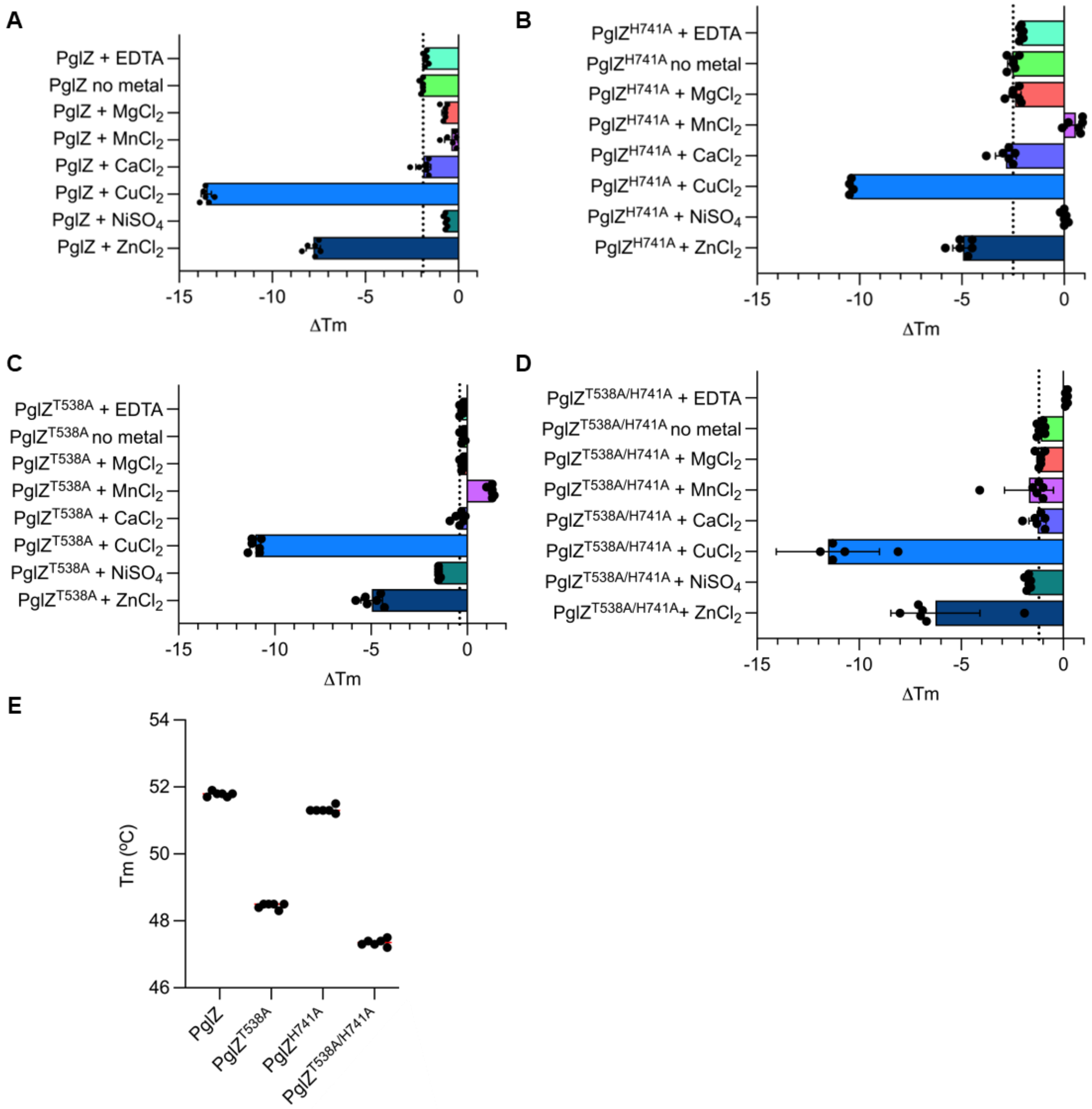

Supplementary Figure S8. Thermal shift assay of *E. fergusonii* PglZ WT (**A**) and mutants H741A (**B**), T538A (**C**), and T538A/H741A (**D**), showing metal-dependent thermostability. Mean changes in melting temperature (ΔTm) are plotted by comparison to respective untreated PglZ WT or mutants, which is set at point '0'. (**E**) The melting temperature (Tm) of PglZ WT and mutants. Protein samples were analysed at 5 μM. Black dotted line represents the average melting temperature of the PglZ WT and mutant no metal controls for comparison with the metal-treated samples. Plotted data represent the mean ± SD (6 replicates).

Supplementary Figure S9

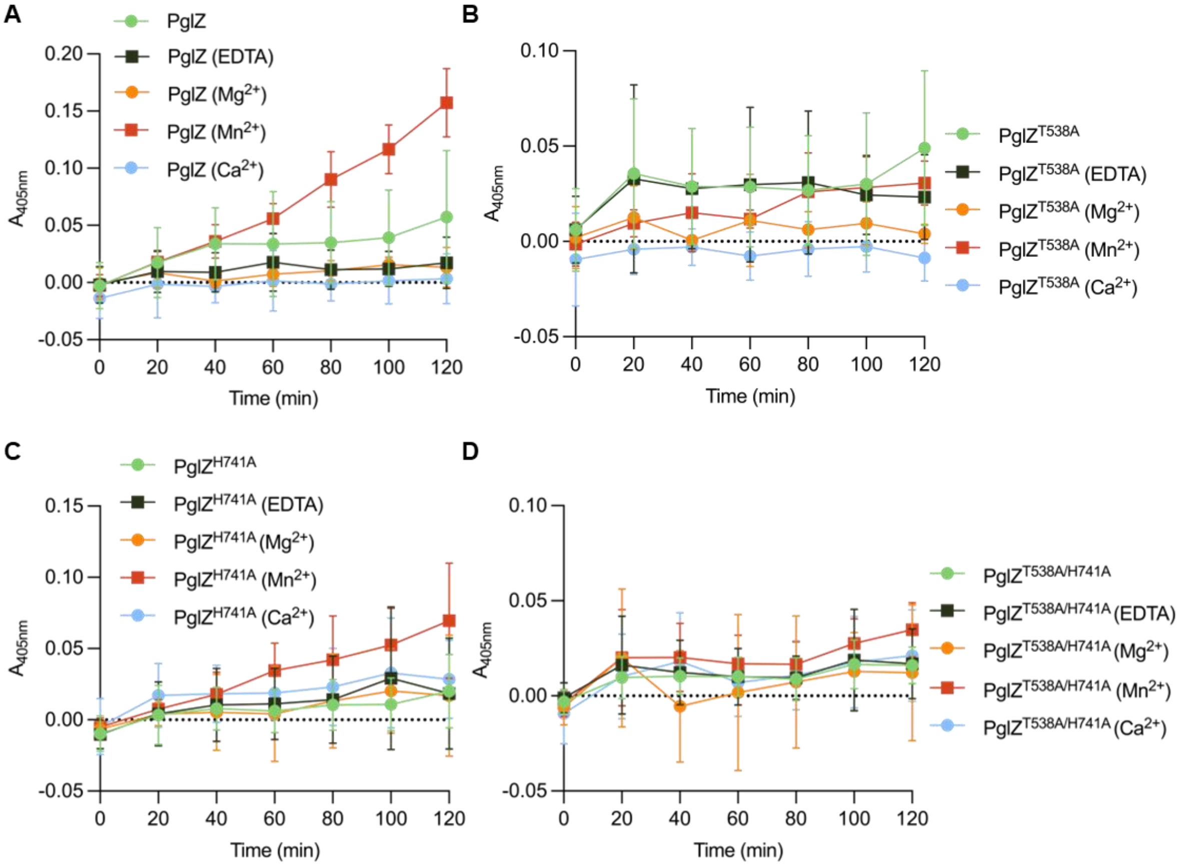

Supplementary Figure S9. *E. fergusonii* PglZ WT (**A**) and mutants T538A (**B**), H741A (**C**), and T538A/H741A (**D**) Bis-*p*NPP phosphodiesterase assays, showing metal-dependent phosphodiesterase activity. Absorbance (A_{405nm}) represents the amount of reaction product *p*-nitrophenyl phosphate. Protein samples were tested at 2 µM in the presence of 2.5 mM Bis-*p*NPP, with or without EDTA and 0.5 mM metal. Control reactions comprised of metal buffer and Bis-*p*NPP in the absence of PglZ, which were set at absorbance '0' for each timepoint. Plotted data represent mean absorbance readings ± SD (9 replicates).

Supplementary Figure S10

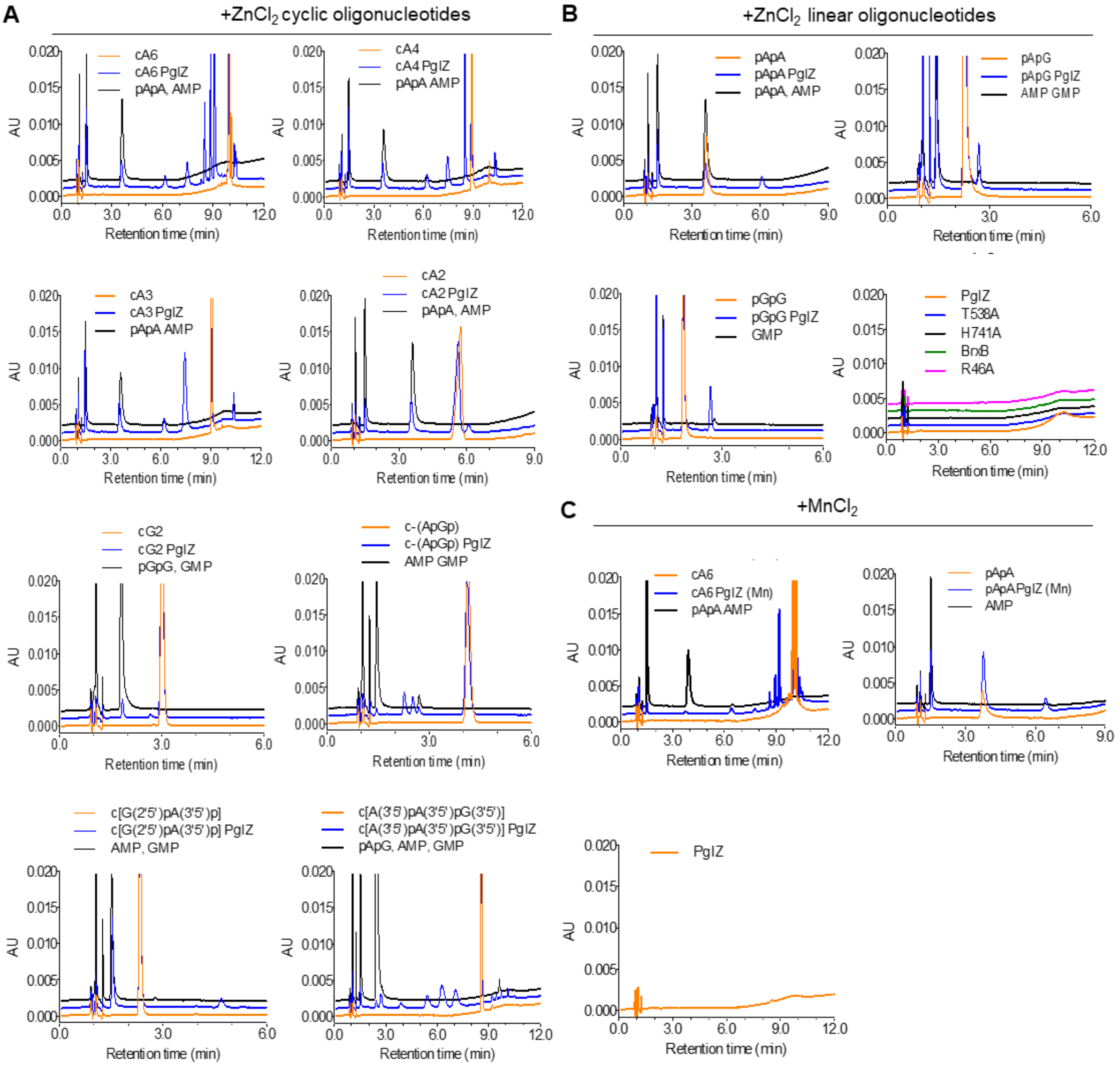

Supplementary Figure S10. *E. fergusonii* PglZ (2 µM) can cleave a range of A and G nucleotides (10 µM) in a zinc- or manganese-dependent manner. (**A**) HPLC analysis of PglZ activity on cyclic oligonucleotides, using $ZnCl_2$ (10 µM). (**B**) HPLC analysis of PglZ activity on linear oligonucleotides, using $ZnCl_2$ (10 µM). (**C**) HPLC analysis of PglZ activity on cA6 and pApA, using $MnCl_2$ (10 µM). Control reactions are comprised of the nucleotide in the absence of protein, and the protein in the absence of nucleotide. Standard mixes are comprised of expected degradation products for each reaction. Presented traces are representative of triplicate data.

Supplementary Figure S11

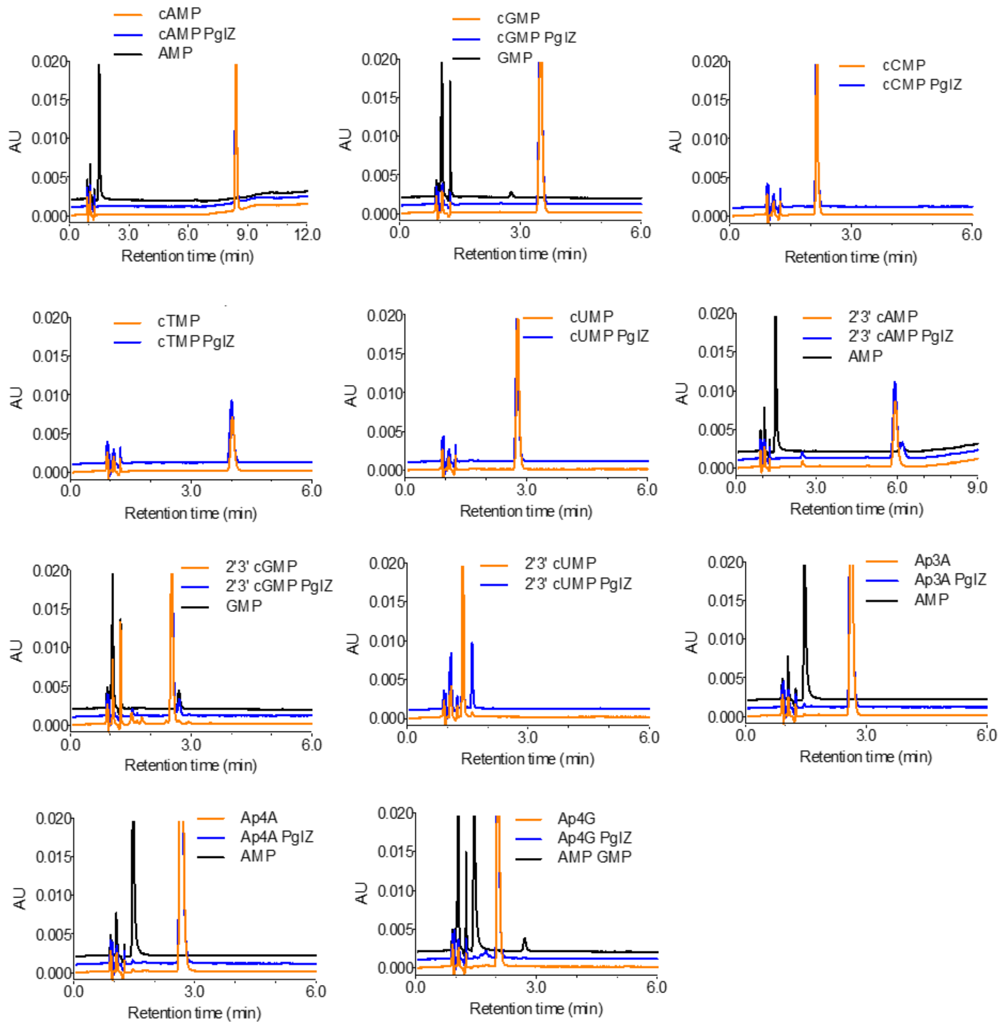

Supplementary Figure S11. *E. fergusonii* PglZ (2 µM) has no cleavage activity on 3′-5′ or 2′-3′ cyclic mononucleotides or dinucleotide polyphosphates (10 µM) in the presence of $ZnCl_2$ (10 µM), analysed by HPLC. Control reactions are comprised of the nucleotide in the absence of protein. Standard mixes are comprised of expected degradation products for each reaction, where possible. Presented traces are representative of triplicate data.

Supplementary Figure S12

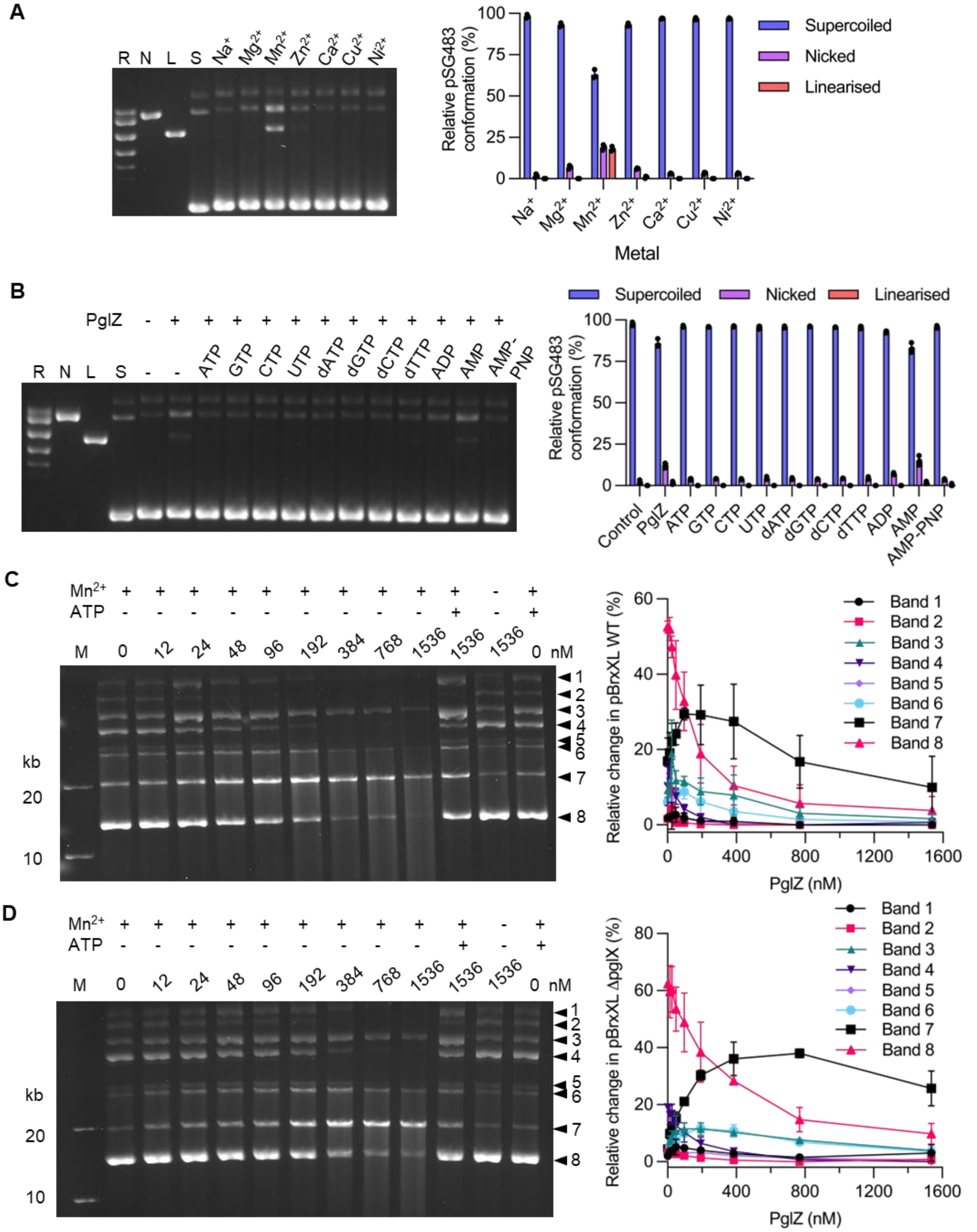

Supplementary Figure S12. Impact of metals, nucleotides, and BREX methylation on *E. fergusonii* PglZ nuclease activity. (**A**) PglZ nuclease activity is maximised in vitro by Mn. PglZ (768 nM) was tested in the presence of 0.5 mM of various metals against supercoiled pSG483 DNA (6 nM) (**B**) A wide range of nucleotides (1 mM) can inhibit PglZ (768 nM) nuclease activity on supercoiled pSG483 DNA (6 nM). PglZ cleavage of pBrxXL WT (**C**) and pBrxXL-Δ*pglX* (**D**) plasmids, which are BREX methylated and non-methylated, respectively. PglZ was titrated against pBrxXL WT (200 ng) and pBrxXL-Δ*pglX* (200 ng) in the presence and absence of $MnCl_2$ (0.5 mM) and ATP (1 mM). Samples were incubated for 1 hour. Control lanes represent supercoiled (S), nicked (N), linear (L), and relaxed (multiple topoisomers; R) plasmid DNA, or contain DNA in the absence of protein. Assays are presented on 1.4% (w/v) or 0.8% (w/v) agarose 1x TAE gels post-stained with ethidium bromide. Assays shown are representative of triplicate experiments. Data points and error bars represent the mean ± SD of triplicate data.

Supplementary Figure S13

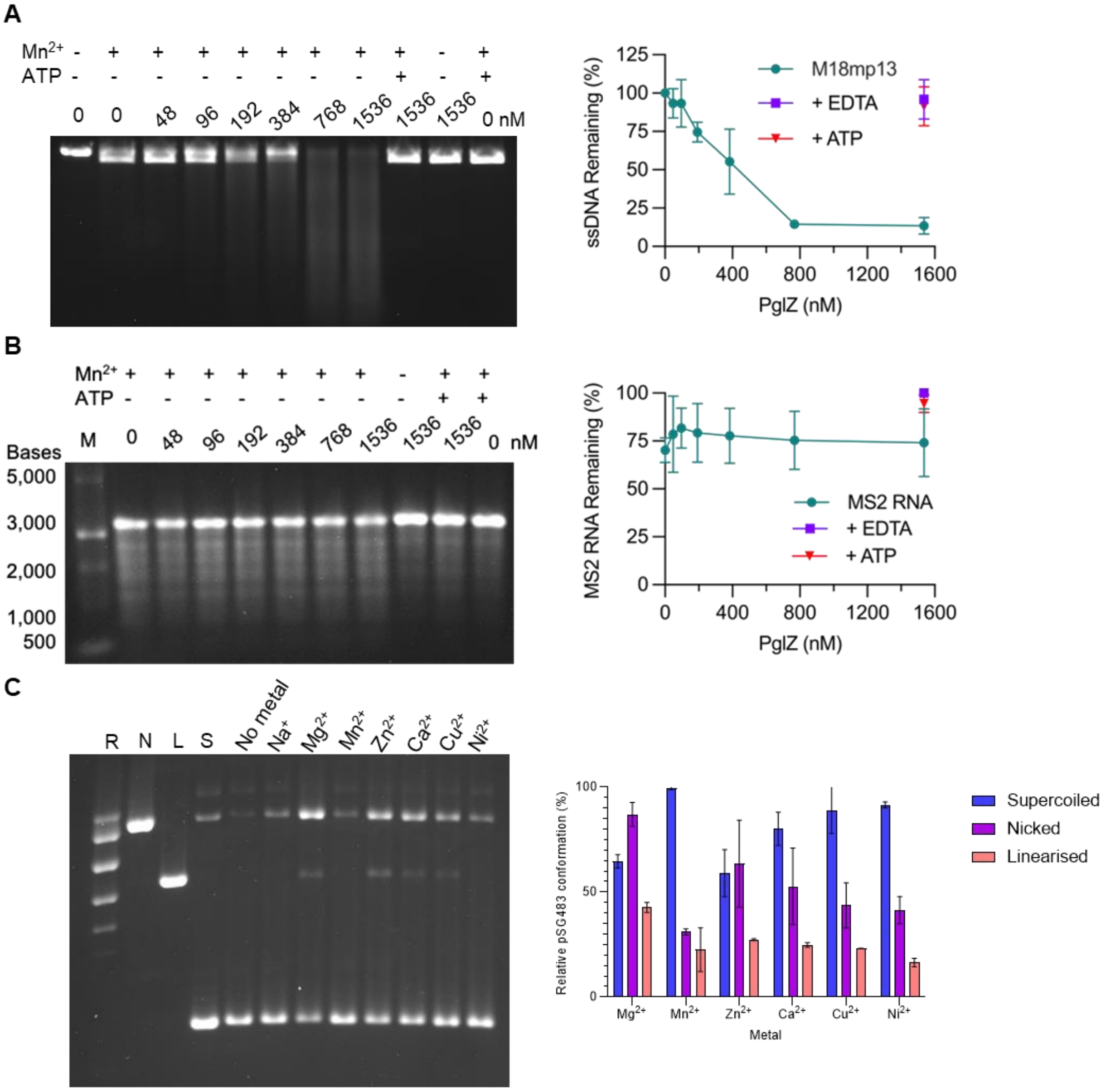

Supplementary Figure S13. *E. fergusonii* PglZ can cut a range of nucleic acids, and *Salmonella* PglZ also demonstrates nuclease activity. (**A**) PglZ (768 nM) cleaves M18mp13 ssDNA (6 nM) in the presence of Mn (0.5 mM). (**B**) PglZ (768 nM) is not able to cleave phage MS2 RNA (6 nM). (**C**) *Salmonella* PglZ nicks and linearises supercoiled pSG483 DNA (6 nM) in a metal-dependent manner. Metal was supplied at 0.5 mM and samples were incubated for 1 hour. Control lanes represent supercoiled (S), nicked (N), linear (L), and relaxed (multiple topoisomers; R) plasmid DNA, or contain DNA in the absence of protein. Assays are presented on 1.4% (w/v) or 0.8% (w/v) agarose 1x TAE gels post-stained with ethidium bromide. Assays shown are representative of triplicate experiments. Data points and error bars represent the mean ± SD of triplicate data.

Supplementary Figure S14

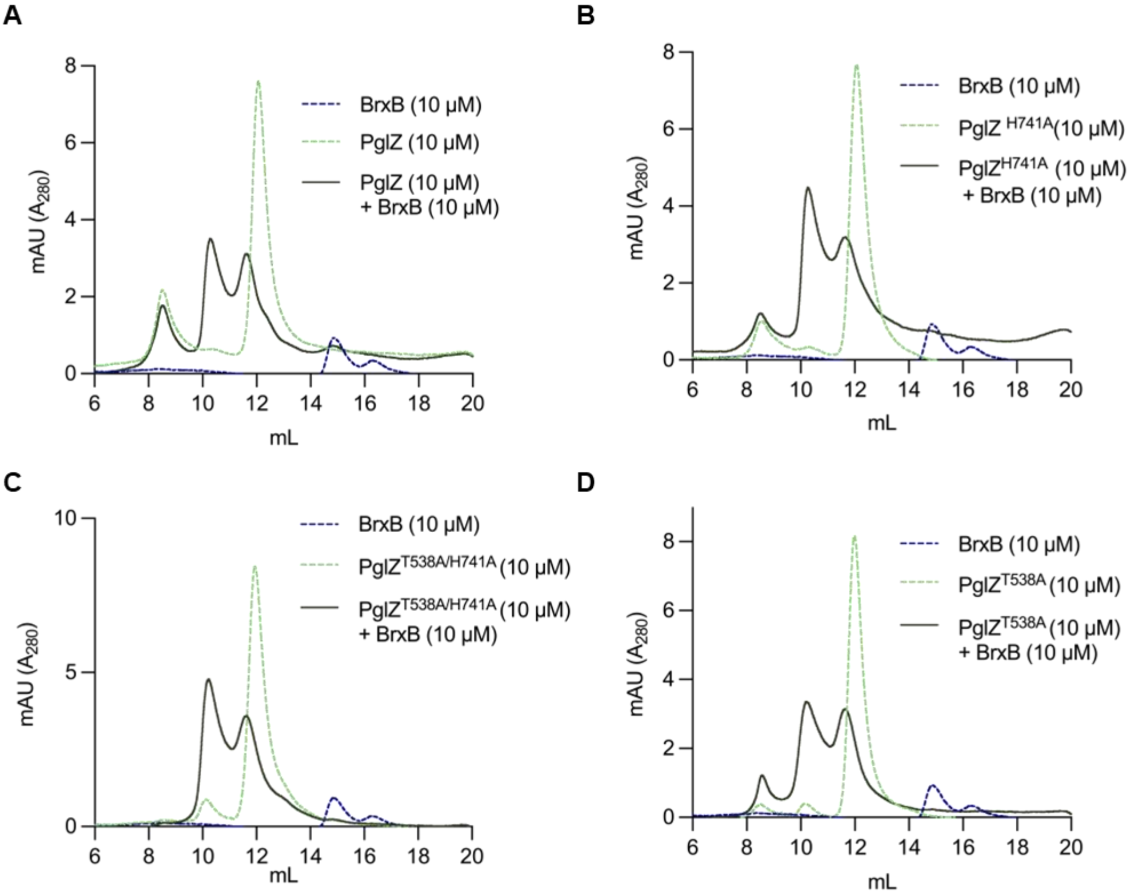

Supplementary Figure S14. *E. fergusonii* PglZ WT (**A**) and mutants H741A (**B**), T538A/H741A (**C**), and T538A (**D**) interact similarly with BrxB WT, analysed by analytical SEC (S200i). Samples of BrxB (10 μM) with equimolar PglZ WT and mutants were made up to 100 μl and pre-incubated for 15 min prior to loading on the S200i. The elution profile of PglZ incubated with BrxB is shown as a dark green solid line. Control elution profiles of PglZ alone (light green dashed line) and BrxB alone (dark blue dashed line) are also shown.

Supplementary Table S1. Plasmids used in this study.

PLASMID	NOTES	SYNTHESIS	REFERENCE
p2HR-T	Ap^R		Addgene #29718
p2HR-T-brxB	*Salmonella* 2HR-T His-strep-BrxB. Ap^R	Genscript	This study
pCDF DUET1	Sp^R		Novagen
pCOLA DUET1	Km^R		Novagen
pET DUET1	Ap^R		Novagen
pET15b.PglZ^{Aci}-TST	*Acinetobacter* PglZ. C-terminal Twin-Strep tag (thrombin cleavable). Ap^R.	Gibson subcloning	This study
pET24d.BrxB^{Aci}	*Acinetobacter* BrxB, untagged. Km^R	Gibson subcloning	This study
pSALMB	*Salmonella* pSAT-LIC-*brxB*. Ap^R	Genscript	This study
pSALMZ	*Salmonella* pSAT-LIC-*pglZ*. Ap^R	Genscript	This study
pSALM-BREX	*Salmonella* pCOLA DUET1 *brxA*, *brxB*, *brxC*, *pglX* (MCS1) and *pglZ*, *brxL* (MCS2). Km^R	Genscript	This study
pSG483	Ap^R		Beck *et al*., 2024
pSG483-BREX KO	Ap^R	Genscript	This study
pTRB444	*E. fergusonii* pSAT1-LIC-*brxB*. Ap^R	Genscript	This study
pTRB473	*E. fergusonii* pSAT1-LIC-*pglZ*. Ap^R	Genscript	This study
pTRB563	*E. fergusonii* pBrxXL WT. Cm^R	Golden gate assembly	Picton *et al*., 2021
pTRB564	*E. fergusonii* pBrxXL-*ΔpglX*. Cm^R	Golden gate assembly	Picton *et al*., 2021
pTRB710	*Salmonella* pET DUET1 His-SUMO-*brxB* and *pglZ*. Ap^R	Genscript	This study
pTRB724	*E. fergusonii* pSAT1-LIC-*brxB* E47A. Ap^R	Genscript	This study
pTRB725	*E. fergusonii* pSAT1-LIC-*brxB* S133A. Ap^R	Genscript	This study
pTRB726	*E. fergusonii* pSAT1-LIC-*brxB* R46A. Ap^R	Genscript	This study
pTRB727	*E. fergusonii* pSAT1-LIC-*brxB* W135A. Ap^R	Genscript	This study
pTRB728	*E. fergusonii* pSAT1-LIC-*brxB* E89A. Ap^R	Genscript	This study
pTRB729	*E. fergusonii* pSAT1-LIC-*pglZ* T538A. Ap^R	Genscript	This study
pTRB730	*E. fergusonii* pSAT1-LIC-*pglZ* H741A. Ap^R	Genscript	This study
pTRB744	*E. fergusonii* pBrxXL *brxB* W135A. Cm^R	Genscript	This study
pTRB745	*E. fergusonii* pBrxXL *pglZ* H741A. Cm^R	Genscript	This study
pTRB746	*E. fergusonii* pBrxXL *brxB* E47A. Cm^R	Genscript	This study
pTRB747	*E. fergusonii* pBrxXL *brxB* E89A. Cm^R	Genscript	This study
pTRB748	*E. fergusonii* pBrxXL *brxB* S133A. Cm^R	Genscript	This study
pTRB749	*E. fergusonii* pBrxXL *brxB* R46A. Cm^R	Genscript	This study
pTRB750	*E. fergusonii* pBrxXL *pglZ* T538A. Cm^R	Genscript	This study
pTRB758	*Salmonella* pCOLA DUET1 *brxA* and *brxL*. Km^R	Genscript	This study

pTRB759	*Salmonella* pCDF DUET1 *brxC* and *pglX*. Sp^R	Genscript	This study
pTRB763	*E. fergusonii* pSAT1-LIC-*pglZ* T538A/H741A. Ap^R	Genscript	This study
pTRB766	*E. fergusonii* pBrxXL *pglZ* T538A/H741A. Cm^R	Genscript	This study
